# Viral Vector-Based Dissection of Marmoset GFAP Promoter in Mouse and Marmoset Brains

**DOI:** 10.1371/journal.pone.0162023

**Published:** 2016-08-29

**Authors:** Yoichiro Shinohara, Ayumu Konno, Nobutaka Takahashi, Yasunori Matsuzaki, Shoji Kishi, Hirokazu Hirai

**Affiliations:** 1 Department of Neurophysiology & Neural Repair, Gunma University Graduate School of Medicine, Maebashi, Gunma, Japan; 2 Department of Ophthalmology, Gunma University Graduate School of Medicine, Maebashi, Gunma, Japan; 3 Research Program for Neural Signalling, Division of Endocrinology, Metabolism and Signal Research, Gunma University Initiative for Advanced Research, Maebashi, Gunma, Japan; Osaka University Graduate School of Medicine, JAPAN

## Abstract

Adeno-associated virus (AAV) vectors are small in diameter, diffuse easily in the brain, and represent a highly efficient means by which to transfer a transgene to the brain of a large animal. A major demerit of AAV vectors is their limited accommodation capacity for transgenes. Thus, a compact promoter is useful when delivering large transgenes via AAV vectors. In the present study, we aimed to identify the shortest astrocyte-specific GFAP promoter region that could be used for AAV-vector-mediated transgene expression in the marmoset brain. The 2.0-kb promoter region upstream of the *GFAP* gene was cloned from the marmoset genome, and short promoters (1.6 kb, 1.4 kb, 0.6 kb, 0.3 kb and 0.2 kb) were obtained by progressively deleting the original 2.0-kb promoter from the 5’ end. The short promoters were screened in the mouse cerebellum in terms of their strength and astrocyte specificity. We found that the 0.3-kb promoter maintained 40% of the strength of the original 2.0-kb promoter, and approximately 90% of its astrocyte specificity. These properties were superior to those of the 1.4-kb, 0.6-kb (20% promoter strength) and 0.2-kb (70% astrocyte specificity) promoters. Then, we verified whether the 0.3-kb GFAP promoter retained astrocyte specificity in the marmoset cerebral cortex. Injection of viral vectors carrying the 0.3-kb marmoset GFAP promoter specifically transduced astrocytes in both the cerebral cortex and cerebellar cortex of the marmoset. These results suggest that the compact 0.3-kb promoter region serves as an astrocyte-specific promoter in the marmoset brain, which permits us to express a large gene by AAV vectors that have a limited accommodation capacity.

## Introduction

Astrocytes express various receptors and transporters for neurotransmitters and are actively involved in neuronal processing by modulating local synaptic functions: impairment of astrocytes affects the synaptic function, leading to associated behavioral defects. For example, in the cerebellar cortex, genetic removal of α-amino-3-hydroxy-5-methyl-4-isoxazolepropionic acid (AMPA)-type glutamate receptors expressed on Bergmann glial appendages results in impairments in fine motor coordination [1]. On the other hand, mutant mice lacking type-1 cannabinoid (CB1) receptors specifically in hippocampal astrocytes failed to demonstrate long-term depression of CA3-CA1 synapses and exhibited impairments in spatial working memory [2]. Moreover, astrocytes have been proposed to underlie the pathological states of various neurological diseases, such as spinocerebellar ataxia (SCA) type 1 [3, 4], SCA type 7 [5, 6] and multiple sclerosis [7]. Thus, selective gene modification of astrocytes is a useful approach for exploring the pathophysiological roles of astrocytes on a molecular basis and could be a therapeutic intervention for diseases associated with astrocyte impairment.

Vectors derived from adeno-associated virus (AAV) and lentivirus are valuable for efficiently introducing transgenes into astrocytes [8–10]. AAV vectors have smaller diameters (~20 nm) than lentiviral vectors (~100 nm), and diffuse easily into the brain parenchyma, leading to transduction across large areas of brain tissue [8]. Therefore, AAV vectors are preferable to lentiviral vectors as mediators of gene transfer to large areas of the brain in the case of large animals such as primates. A major drawback of AAV vectors, however, is a limited capacity to accommodate a transgene. While lentiviral vectors can accommodate a transgene as large as 8 kb, AAV vectors have a capacity of only 4.7 kb, including a promoter sequence [11]. Hence, it is difficult to express a 3-kb transgene with a 2-kb promoter. Generally, cell type-specific promoters are large in size because they contain a region that restricts the promoter activity to a specific cell population. Thus, a compact astrocyte-specific promoter is valuable for AAV vector-mediated transgene expression.

Common marmosets (*Callithrix jacchus*) have several advantages over macaques because they (1) are smaller and easier to handle, (2) have a higher efficiency in producing offspring and (3) do not carry fatal zoonotic diseases, such as the herpes b virus [12]. Marmosets can use their fingers to pinch food, quickly move upward and downward between tree limbs, live in family groups and communicate with each other through various vocalizations. These characteristics suggest that marmosets are superior to mice for examining a variety of brain functions, such as motor control, language and social behavior. Thus, marmosets have been increasingly used in the field of neuroscience. AAV vector-mediated transfer of various transgenes into astrocytes of the large marmoset brain *in vivo* allows us to clarify the pathophysiological roles that astrocytes play in the brain and, as a result, further extend the astrocyte-specific transgene expression capabilities available for gene therapies. In the present study, we aimed to develop a compact, astrocyte-specific promoter that could be used to transfer a large transgene (more than 3 kb) in combination with AAV vectors in the marmoset brain.

## Materials and Methods

### Animals

All procedures regarding animal care and treatment were performed in accordance with the institutional and national guidelines and were approved by the Institutional Committee of Gunma University (No. 12–013; 15–038). Wild-type C57BL/6 mice aged between 3 and 4 weeks and two young adult common marmosets (*Callithrix jacchus*, H033: male, body weight (BW) 331g, H039: male, BW 343g) were used in this study. Mice were bred and housed at the Gunma University. Common marmosets were purchased through CLEA Japan (Tokyo, Japan) and bred at the Gunma University Bioresource Center. All animals were bred for research purpose. Common marmosets were kept in individual primate cages (375 mm × 550 mm × 762 mm) and maintained in rooms under controlled temperature (26 to 28°C), humidity (30 to 70%) on a 12-h light and 12-h dark cycle. We daily gave water *ad libitum* and 40 to 50 g of soaked monkey chow (CMS-1, CLEA Japan) with vitamin supplements, fresh fruit, vegetables, boiled chicken or milk powder. Wood branches and iron perches were placed in each cage for environmental enrichment. Animals were daily monitored to assess their health and symptoms, including a food intake, diarrhea, weight loss and trauma. Handling all animals was based on the Guide for the Care and Use of Laboratory Animals (8^th^ edition). We made every effort to minimize animal suffering and reduce the number of animals used in the present study.

### Construction of expression plasmid with cjGFAP promoters

The marmoset GFAP (*Callithrix jacchus* GFAP; cjGFAP) promoter region (Accession number: BR001393) upstream of the *Gfap* gene was amplified by polymerase chain reaction (PCR) of marmoset genomic DNA. To obtain the original 2-kb cjGFAP promoter region effectively, we performed nested PCR with 2 pairs of primers: cjGFAP (Nest)-F (forward primer), 5’-AGGGTCAGATGTGACTAGAGCC-3’ / cjGFAP (Nest)-R (reverse primer), 5’-GACGATTGTTGGACAGTGAG-3’ and cjGFAP (2.0)-F, 5’- CGGAACGCGTATGTGGGAAGATTGCTTGAGCCTAG-3’ / cjGFAP (2.0)-R, 5’-GTCGAATTCCCTGCCCTGGCTCTGCTTGC-3'. The shorter GFAP promoters were produced by 5’ deletion of the original 2-kb GFAP promoter using the following primers: cjGFAP (1.6)-F, 5’-CGGAACGCGTCGTGCCCACTGAATGACTCACC-3’, cjGFAP (1.4)-F, 5’-CGGAACGCGTGGCGCCACCGGCGGTGGAGAAC-3’, cjGFAP (0.6)-F, 5’- CGAACGCGTATCAAAAAGCTGGAAGGCAG-3’, cjGFAP (0.3)-F, 5’- CGGAACGCGTGTGGTCCAACCAACCCTTCTTGAC-3’ or cjGFAP (0.2)-F, 5’- CGGAACGCGTTGCCTCATGCAGGAGTTGGCGTG-3’ and cjGFAP (2.0)-R, 5’-GTCGAATTCCCTGCCCTGGCTCTGCTTGC-3'. The 0.3-kb mouse GFAP (mGFAP) (Accession number: BR001394) was amplified by PCR of mouse genomic DNA using mGfa2 (0.3)-F, 5’-CCGACGCGTGTGGGTCTTCATGCTTGACA-3’ and mGFAP (0.3)-R, 5’-GGAATTCCCTGCCCTGCCTCTGCTG-3’. The 0.3-kb human GFAP (hGFAP) (Accession number: BR001395) was amplified by PCR of human genomic DNA using hGFAP (0.3)-F, 5’- CGACGCGTTTCTTGACCCACCTTCCTAGAG-3’ and hGFAP (0.3)-R, 5’- GGAATTCCCTGCTCTGGCTCTGCTCG-3’. cjGFAP promoters ≤2.0 kb and the 0.3-kb mouse and human GFAP promoters were inserted into the MluI/EcoRI-digested site of the pCL20c-GFP lentiviral vector plasmid. The 0.3-kb cjGFAP promoter was inserted into XhoI/AgeI-digested pAAV-GFP AAV vectors. Proper insertion of the promoters into the viral plasmids was verified by DNA sequencing.

### Virus preparation

Vesicular stomatitis virus glycoprotein (VSV-G)-pseudotyped lentiviral vectors were produced as previously described [13]. We transfected a mixture of four plasmids, consisting of pCAGkGP1R, pCAG4RTR2, pCAG-VSV-G and pCL20c/GFAP-GFP, into HEK293T cells using the calcium phosphate precipitation method. The supernatant containing the viral particles was harvested at 48 h post-transfection. After ultracentrifugation of the supernatant, the precipitated virus particles were resuspended in 70 μl of phosphate-buffered saline (-) (PBS). The lentiviral titers were determined using the comparative threshold cycle (*C*_T_) method by quantitative real-time PCR and with the following procedure: genomic RNA was isolated from 2 μl of viral solutions using an RNeasy Mini Kit (Qiagen, Hilden, Germany), which was subsequently reverse-transcribed using the ReverTra Ace qPCR RT Master Mix with gDNA Remover (Toyobo, Tokyo, Japan). The amounts of the synthesized cDNAs were quantified by quantitative real-time PCR using a protocol of 95°C for 1 min, 95°C for 15 s and 60°C for 30 s, 40 cycles (Takara Thermal Cycler Dice TP800, Takara Bio, Shiga, Japan) using THUNDERBIRD^®^ SYBR^®^ qPCR Mix (Toyobo) and the following primers: EGFP-F, 5’-TGG TGC AGA TGA ACT TCA GGG-3’ and EGFP-R, 5’-GTA AAC GGC CAC AAG TTC AGC-3’. The lentiviral solution was stored at 4°C and used within 2 weeks.

Recombinant single-strand AAV9 vectors were produced by transfection of HEK293T cells (Thermo Fisher Scientific, Waltham, MA) with pAAV/cjGFAP-GFP, pAAV2/9 (kindly provided by Dr. J. Wilson) and a helper plasmid (Stratagene, La Jolla, CA) as previously described [14]. The viral particles were purified using ammonium sulfate precipitation and iodixanol continuous gradient centrifugation as previously described. The genomic titer of the purified AAV9 vectors was determined by real-time PCR. The pAAV/cjGFAP-GFP plasmids were used for the generation of standard curves of viral genomic titer.

### Injection of viral vectors into mouse and marmoset brains

After deep anesthesia via an intra-peritoneal injection of ketamine (100 mg/kg BW) and xylazine (10 mg/kg BW), mice were placed in a stereotactic frame. For injection into the cerebellum, the skin covering the occipital bone was cut, and a burr hole was made 2 mm caudal from the lambda. The tip of a Hamilton syringe (33 gauge) with an attached micropump (UltraMicroPump II; World Precision Instrument (WPI) Sarasota, FL, USA) was inserted 1.8 mm below the pia mater of the cerebellar vermis. The viral solution (10 μl) was injected at a rate 450 nl/min using a microprocessor-based controller (Micro4; WPI). For injection into the cerebral cortex, the skin over the cerebral hemisphere was cut, and a burr hole was made 1 mm anterior-posterior (A/P), 1 mm medial-lateral (M/P) and 0.8 mm dorsal-ventral (D/V) from the bregma. The viral solution (5 μl) was injected into the cerebral cortex at a rate of 225 nl/min using a Micro4 (WPI). The syringe was left in place for 2 min following the injection. After closing the scalp, the mice were kept on a heating pad until they recovered from the anesthesia. Then, the mice were returned to standard home cages. For injection of AAV9 vectors, each marmoset received an intramuscular injection of ketamine and xylazine (20–25 mg/kg BW and 1.6–2.0 mg/kg BW, respectively) for sedation, and anesthesia was maintained by inhalation of 2.0–2.5% isoflurane to minimize their suffering and distress. The marmoset was then placed in a stereotactic frame, and heart rate and oxygen saturation were monitored throughout the procedure. A burr hole was made for cerebellar injection at 4 mm A/P, 3 mm M/L and 4 mm D/V from the external occipital protuberance. A second burr hole was made for cerebral injection at 0 ± 3 mm A/P, 2 mm M/L and 2.5 mm D/V from the bregma. Fifty (cerebellum) and 10 (cerebrum) μl of AAV9 vector suspension (2.0×10^12^ vector genomes/ml) was injected at rates of 5 μl/min and 1 μl/min, respectively. The syringe was left in place for 2 min after injection. Then the scalp was sutured, its vital signs were monitored until it recovered from the anesthesia. After awaking, it was returned to a standard home cage. A humane endpoints were in place during the animal experiments as the following indicators: severe pain, severe distress, suffering or impending death, at which conditions, marmosets were humanely euthanized. However, the operated marmosets showed only a slight ataxia for a couple of days after the viral injection, and returned quickly to their original condition thereafter. Thus, no mortality was observed.

### Histological analysis and immunohistochemistry

The mice were sacrificed 7 days after viral injection. These deeply anesthetized mice were perfused intracardially with 4% paraformaldehyde in 0.1 M phosphate buffer. The whole brains were immersed in 4% paraformaldehyde in 0.1 M phosphate buffer. The cerebellum and cerebrum were cut into 50-μm sagittal sections using a microtome (Leica VT1000 S; Leica Microsystems, Wetzlar, Germany). The slices were blocked with PBS containing 2% normal donkey serum, 0.1% Triton X-100, and 0.05% NaN_3_ (blocking solution) and then incubated overnight at 4°C in the following primary antibodies: rabbit polyclonal anti-GFP (1:1000; Rb-Af2020; Frontier Institute, Hokkaido, Japan) and mouse monoclonal anti-S100 (1:1000; S2532; Sigma-Aldrich, St. Louis, MO, USA) for cerebellar slices or rat monoclonal anti-GFP (1:1000; 04404–84; Nacalai, Kyoto, Japan), rabbit polyclonal anti-GFAP (1:200; RB-087-A0; Thermo Fisher Scientific, Waltham, MA, USA) and mouse monoclonal anti-NeuN (1:1000; MAB377, Merk Millipore, Billerica, MA, USA) for cerebral sections. After washing two and three times with 0.5% and 0.1% Triton X-100 in PBS, respectively, at room temperature, the slices were incubated in blocking solution for 2 h at room temperature in the following secondary antibodies: Alexa Fluor 488 donkey anti-rabbit IgG (1:1000; Thermo Fisher Scientific, Waltham, MA) and Alexa Fluor 568 donkey anti-mouse IgG (1:1000; Thermo Fisher Scientific) for cerebellar slices or Alexa Fluor 488 donkey anti-rat IgG (1:1000; Thermo Fisher Scientific), Alexa Fluor 568 donkey anti-rabbit IgG (1:1000; Thermo Fisher Scientific) and Alexa Fluor 680 donkey anti-mouse IgG (1:1000; Thermo Fisher Scientific) for cerebral sections. After the secondary antibody reaction, Nissl bodies of the cerebellar slices were stained with NeuroTrace 640/660 (1:200; Thermo Fisher Scientific) in PBS for 1 h at room temperature. Immunostained sections were mounted in ProLong Gold or Diamond antifade reagents (Thermo Fisher Scientific).

Fluorescence images were obtained on a fluorescence microscope (VB-7010 or BZ-X700; Keyence, Osaka, Japan) or a confocal laser-scanning microscope (LSM 5 or LSM 880; Carl Zeiss, Oberkochen, Germany).

The marmosets were sacrificed 2 or 4 weeks after AAV9 vector injection. The marmosets were anesthetized with a mixture of ketamine and xylazine, and isoflurane. These deeply anesthetized marmosets were perfused intracardially with 1×PBS (pH 7.4) and 4% paraformaldehyde in 0.1 M phosphate buffer. Cerebellar and cerebral sections of 100-μm thickness were obtained using a procedure similar to that used for the mice, and marmoset sections were immunostained with the same antibodies used for the mouse sections.

### Quantification of GFP intensity in mouse cerebellum

To measure the GFP fluorescence intensity, 15 sections from 5 mice (3 sections/mouse) for each promoter were randomly selected, except for the original 2.0-kb cjGFAP promoter, for which 36 sections from 12 mice (3 sections/mouse) were selected. The GFP fluorescence images of those sections were captured using a confocal microscope and the same settings. Then, the outline of the cerebellar section was traced, and the fluorescence intensity in the enclosed areas was measured using ImageJ. The background intensity was subtracted from the fluorescence intensity. The averaged GFP fluorescence intensity of sections treated with lentiviral vectors carrying the 2.0-kb cjGFAP promoter was taken as 100%, and the relative values were plotted on a graph.

### Assessment of the astrocyte specificity of the promoters

We assessed the astrocyte specificity of the promoters by measuring the ratio of GFP-expressing astrocytes to all GFP-expressing cells. We counted more than 500 GFP-positive cells in 9 slices (3 slices/mouse, n = 3 mice), among which the number of GFP/S100 (or GFAP) double-positive astrocytes were examined. The ratio was determined by dividing the number of GFP/S100 (or GFAP) double-positive astrocytes by the total number of GFP-positive cells. Similarly, neuronal leakage of the promoters was assessed by calculating the ratio of GFP/NeuN (or GFP/Nissl substance) double-positive neurons to total GFP-positive cells. GFP-positive cells that failed to show immunolabeling for S100, GFAP, NeuN or NeuroTrace were classified as unknown.

### Statistical analysis

Significant differences were analyzed using one-way analysis of variance (ANOVA) followed by Tukey’s post hoc test. Statistical analyses were performed using the R software statistical package (www.r-project.org). Data are expressed as the mean ± SEM.

## Results

### Deletion constructs of the GFAP promoter

**Fig 1:**
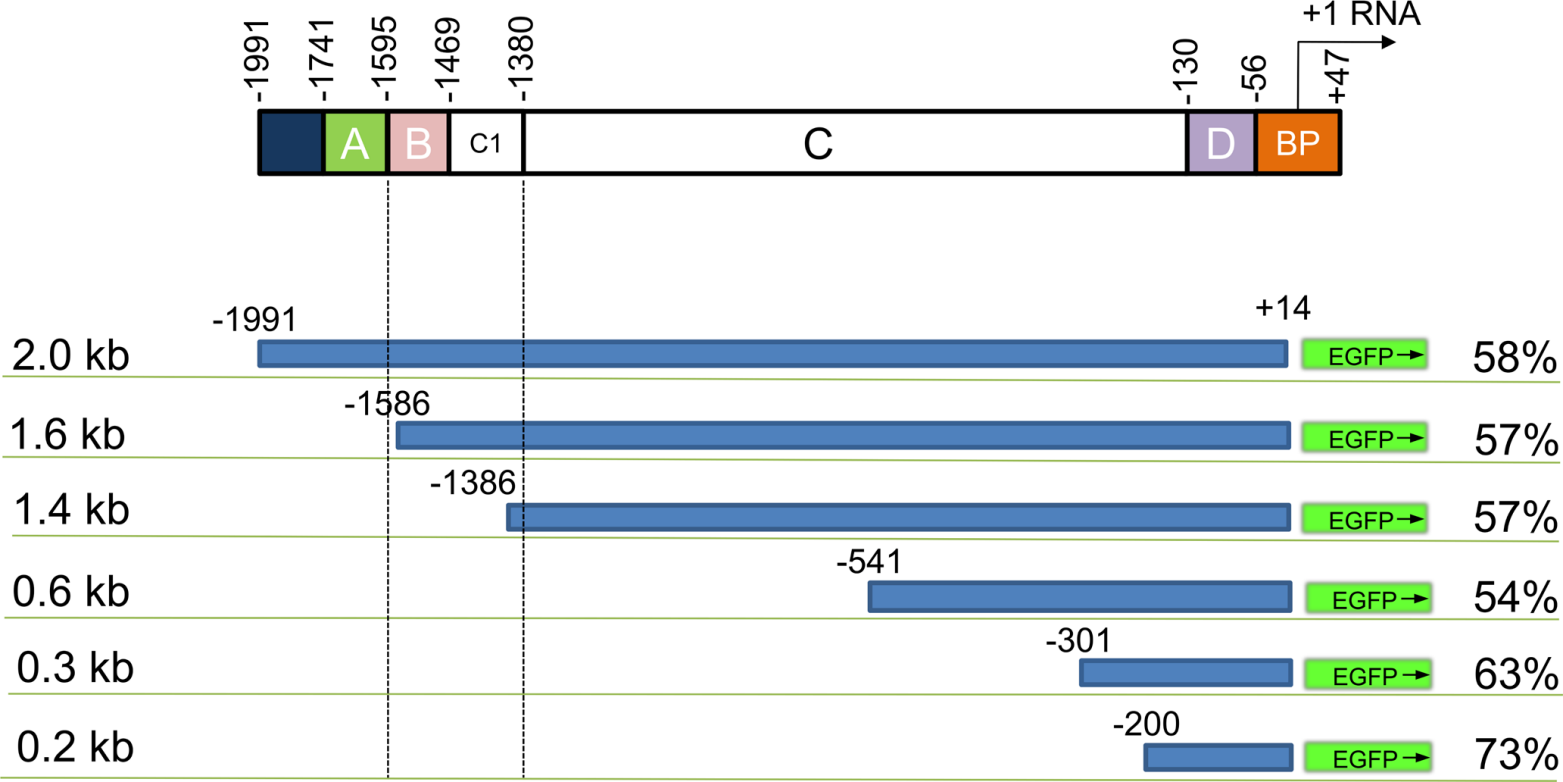
Schema depicting various lengths of the marmoset GFAP promoters examined in this study. The longest GFAP promoter (2.0 kb) spanned from -1991 to +14 bp relative to the transcription start site [18]. The shorter promoters ranged from 0.2 kb to 1.6 kb and included different 5’ deletion. The percentage shown next to each promoter construct is the homology of the marmoset promoter with the mouse promoter. BP; basal promoter.

Previous transgenic studies have shown that the 2.2-kb human GFAP promoter extending 2.2 kb upstream of the RNA start site (bp +1) serves as an astrocyte-specific promoter in the mouse brain [15]. Furthermore, promoter activity, as assessed by chloramphenicol acetyltransferase activity in U251 cells (a human glioma cell line that strongly expresses GFAP), remained almost unchanged between 2.2 kb and 1.7 kb [16]. We cloned a homologous 2.0-kb GFAP promoter region (from +14 to -1991) that displayed 58% and 88% sequence similarity with the mouse and human regions, respectively. The shorter promoter constructs with sizes of 1.6, 1.4, 0.6, 0.3 and 0.2 kb were produced by 5’ deletion of the original marmoset-derived GFAP promoter (cjGFAP promoter) (Fig 1). The B region and C1 segment were shown to carry various transcription factor-binding sites [17]. The 1.6-kb cjGFAP promoter retained all of those transcription factor-binding sites, whereas the cjGFAP promoters ≤1.4 kb lacked the B and C1 regions and, therefore, were devoid of these transcription factor-binding sites.

### Assessment of the promoter strength of the cjGFAP promoter fragments

**Fig 2:**
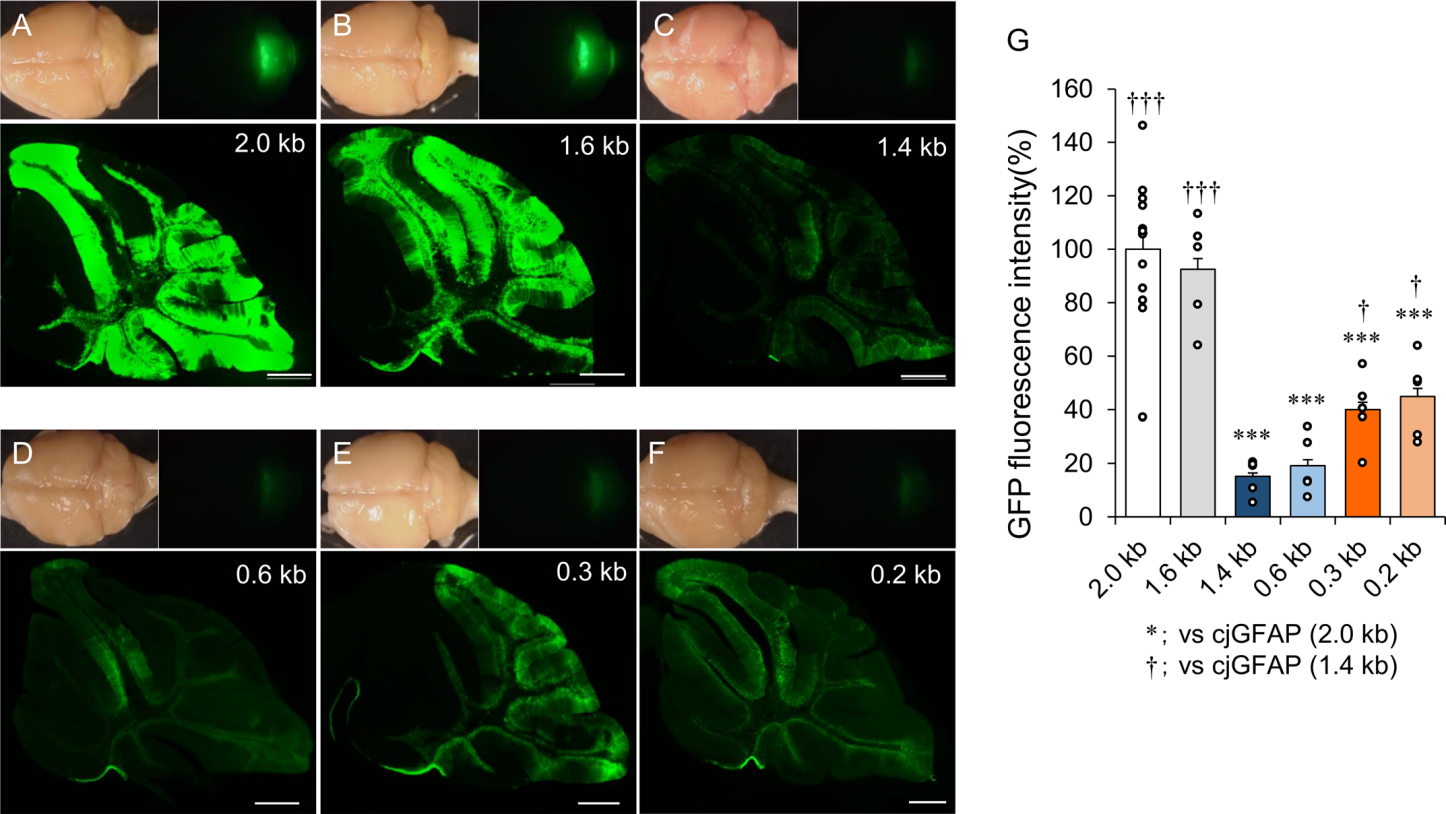
Mouse cerebella lentivirally expressing GFP under the control of cjGFAP promoters of different lengths. (A–F) Upper small panels are bright field (left) and GFP fluorescence (right) images of whole brains. Lower panels show GFP fluorescence images of cerebellar sagittal sections. The promoter lengths used to drive GFP expression are shown in the upper right corner. Scale bars, 500 μm. (G) Quantitative analysis of GFP fluorescence intensity. The fluorescence intensity on sagittal sections of the viral vector-treated cerebellar vermis was measured using ImageJ software. Between 5 and 12 mice in each group were used for the analysis, and the intensities relative to the control 2.0-kb cjGFAP promoter are presented. Small circles in each lane are individual measurements. Asterisks and daggers indicate statistically significant differences compared with mice expressing GFP under the control of the 2.0-kb promoter (asterisks) or the 1.4-kb promoter (daggers), as determined by one-way ANOVA followed by Tukey’s post hoc test, ***p<0.001, ^†^*p*<0.05 and ^†††^*p*<0.001.

To minimize the number of marmosets used, we screened the promoter strength and astrocyte specificity of the deletion constructs in mouse brains using lentiviral vectors. We opted for lentiviral vectors as they can be prepared more quickly (4 days) and with less effort than AAV vectors (10 days), and were sufficient to transduce the small mouse brain. Lentiviral vectors expressing GFP under the control of the full-length cjGFAP promoter or promoter fragments were produced. These viral vectors were injected into the cerebella of mice aged 3–4 weeks to assess promoter strength, as determined by GFP fluorescence intensity. The injected mice were then sacrificed 1 week after the viral injection. We found that the whole cerebellum in mice treated with lentiviral vectors carrying the 2.0- or 1.6-kb promoter showed markedly brighter GFP fluorescence intensity than those treated with lentiviral vectors carrying the shorter cjGFAP promoters (Fig 2A–2F, upper panels). Examination of the cerebellar sagittal sections confirmed the results of the whole cerebellum analysis: a remarkably stronger GFP fluorescence was observed in sections treated with lentiviral vectors carrying the 2.0- or 1.6-kb cjGFAP promoter compared with the shorter cjGFAP promoters (Fig 2A–2F, lower panels). Notably, the GFP fluorescence intensities of cerebellar sections expressing GFP from the 0.3- and 0.2-kb cjGFAP promoters (Fig 2E and 2F) appeared to be brighter than those from the 1.4- and 0.6-kb cjGFAP promoters (Fig 2C and 2D). A quantitative analysis showed that the GFP fluorescence intensities of cerebellar sections, relative to those under the control of the original 2.0-kb cjGFAP promoter (100 ± 5.9%, n = 12 mice), were 92.5 ± 4.0% (1.6-kb promoter, n = 5 mice), 15.1 ± 1.3% (1.4-kb promoter, n = 5 mice), 19.1 ± 2.2 (0.6-kb promoter, n = 5 mice), 40.0 ± 2.7% (0.3-kb promoter, n = 5 mice) and 44.9 ± 3.0% (0.2-kb promoter, n = 5 mice). The GFP fluorescence intensities for the 0.3- and 0.2-kb cjGFAP promoters were significantly weaker than that for the original 2.0-kb cjGFAP promoter (***p<0.001), but they were approximately 2 times brighter and significantly stronger than those for the 1.4- and 0.6-kb cjGFAP promoters (^†^*p*<0.05) (Fig 2G).

### Assessment of the astrocyte specificity of the cjGFAP promoter fragments

**Fig 3:**
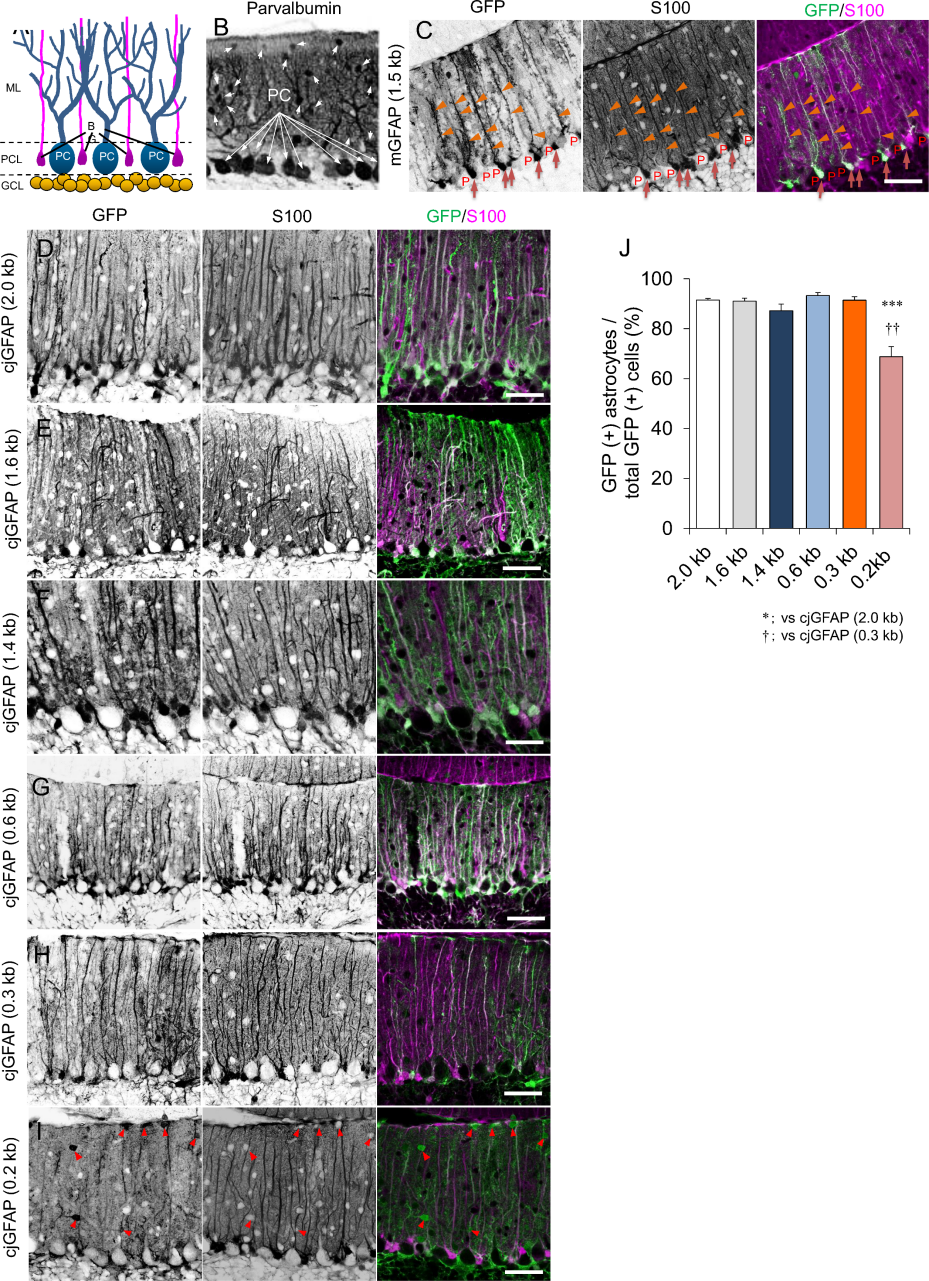
High specificity of the 0.3-kb cjGFAP promoter for astrocytes in the mouse cerebellum. (A) Shema depicting Purkinje cell (PC) and Bergmann glia (BG) in the cerebellar cortex. Cell bodies of Bergmann glia are present in the Purkinje cell layer (PCL) and extend processes into the molecular layer (ML). GCL; Granule cell layer. (B) A cerebellar section immunolabeled for parvalbumin. Arrows indicate molecular layer interneurons. (C) Cerebellar slices lentivirally expressing GFP under the control of the 1.5 kb mouse GFAP promoter were double-immunostained for GFP and S100 (an astrocyte marker). Arrows and arrowheads indicate cell bodies and the processes of Bergmann glia, respectively. GFP expression was observed in Bergmann glia, but not in Purkinje cells (P). (D-I) Cerebellar slices lentivirally expressing GFP under the control of different lengths of the marmoset GFAP promoter were double-immunostained for GFP and S100. Arrows in (I) indicate GFP-positive interneurons in the molecular layer. Scale bars, 50 μm. (J) Quantitative analysis of astrocyte specificity of the marmoset GFAP promoter fragments. More than 500 GFP-positive cells from 3 mice (3 slices/mouse) were randomly selected in each group, and the ratio of S100-labeled astrocytes was determined in these random selections. Asterisks and daggers indicate statistically significant differences compared with the cerebella expressing GFP under the control of the 2.0-kb promoter (asterisks) or the 0.3-kb promoter (daggers), as determined by one-way ANOVA followed by Tukey’s post hoc test, ***p<0.001 and ††*p*<0.01.

Cerebellar cortex is divided into the molecular layer, Purkinje cell layer and granule cell layer (Fig 3A). Purkinje cell somata make the Purkinje cell layer (Fig 3B), where cell bodies of Bergmann glia exist and extend long processes into the molecular layer (Fig 3A). Fig 3C shows a typical example of astrocyte (Bergmann glia)-specific GFP expression in mouse cerebellum that was treated with lentiviral vectors carrying mouse-derived 1.5 kb GFAP promoter: in sharp contrast to absence of GFP expression in Purkinje cells (P, in Fig 3C), robust GFP signal was observed in cell bodies and processes of Bergmann glia, which were co-immunolabeled for S100 (Fig 3C).

To examine the astrocyte specificity of the cjGFAP promoter fragments, cerebellar slices expressing GFP by the cjGFAP promoters (Fig 2A–2F) were double-immunolabeled for GFP and the astrocyte marker S100. Immunohistochemistry showed that 5’ deletion of the cjGFAP promoter did not compromise the astrocyte specificity. The majority of GFP-expressing cells were Bergmann glia (Fig 3D–3I). However, we found a significant number of GFP-expressing interneurons in the cerebella that were transduced by the 0.2-kb cjGFAP promoter (arrowheads, Fig 3I). To quantify the ratios of GFP-positive astrocytes to total GFP-positive cells, we examined more than 500 GFP-positive cells in 9 cerebellar slices from 3 mice and counted the S100-labeled astrocytes. The ratios of GFP-positive astrocytes to total GFP-positive cells were 92.0 ± 0.5% (2.0-kb promoter), 91.8 ± 0.8% (1.6-kb promoter), 89.1 ±1.8% (1.4-kb promoter), 89.7 ±1.1% (0.6-kb promoter), 89.1 ± 1.3% (0.3-kb promoter) and 74.1 ± 3.0% (0.2-kb promoter) (Fig 3J). The ratio was significantly decreased only in cerebellar sections expressing GFP under the control of the 0.2-kb cjGFAP promoter compared with those under the control of the 2.0-kb cjGFAP (***p<0.001) or 0.3-kb cjGFAP (^††^*p*<0.01) promoter. These results suggest that the 0.3-kb cjGFAP promoter shows promise as a compact astrocyte-specific promoter for viral vector-mediated gene expression in the marmoset brain.

### Loss of astrocyte specificity for the 0.3-kb cjGFAP promoter in the mouse cerebrum

**Fig 4:**
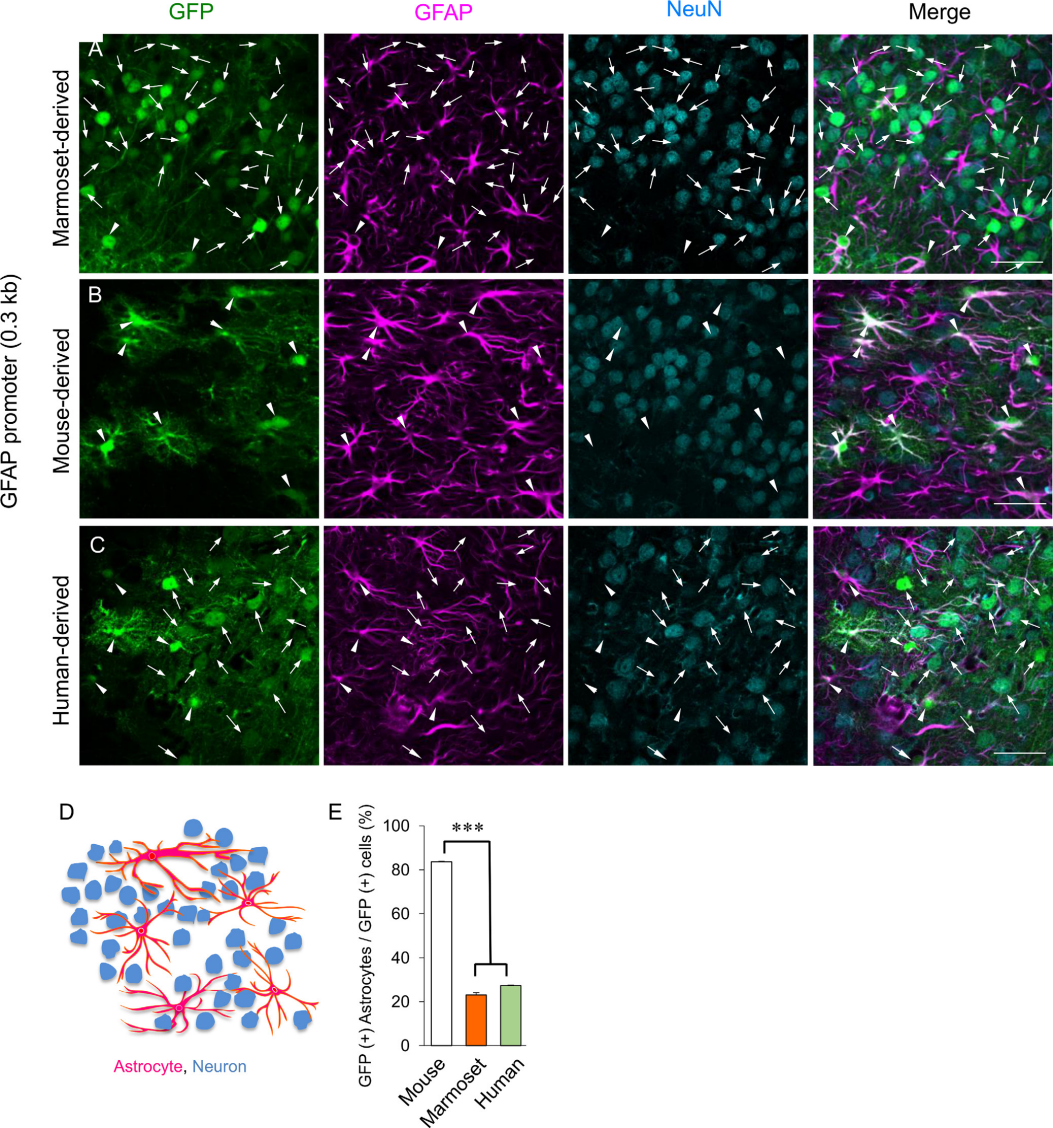
Absence of astrocyte specificity for the marmoset- and human-derived GFAP promoters in the mouse cerebrum. (A-C) Cerebral slices lentivirally expressing GFP under the control of the 0.3-kb marmoset-derived cjGFAP (A), 0.3-kb mouse-derived mGFAP (B) or 0.3-kb human-derived hGFAP (C) promoter were triple-immunostained for GFP (green), GFAP (magenta) and NeuN (a neuronal marker, cyan). Note the predominant expression of GFP in neurons (arrow) by the marmoset- and human-derived promoter, which is in sharp contrast to the astrocyte-specific expression (arrowhead) by the mouse-derived promoter. Scale bars, 50 μm. (D) Schema depicting morphology of neuron and astrocyte in the cerebral cortex. (E) Quantitative analysis of the astrocyte specificity for the cjGFAP, mGFAP and hGFAP promoters. More than 300 GFP-positive cells from 3 mice (3 slices/mouse) were randomly selected, and the ratio of GFAP-labeled astrocytes were determined in these random selections. Asterisks indicate statistically significant differences between the mouse promoter and the marmoset or human promoter, as determined by one-way ANOVA followed by Tukey’s post hoc test, ***p<0.001.

To verify the astrocyte specificity of the 0.3-kb cjGFAP promoter in a different brain region, lentiviral vectors expressing GFP under the control of the 0.3-kb cjGFAP promoter were injected into the mouse cerebral cortex. The cerebral sections obtained 1 week after the viral injection were triple-immunostained for GFP, GFAP and NeuN. The fluorescence microscopic examination clearly showed that a majority of GFP-expressing cells were NeuN-positive and GFAP-negative neuronal cells (arrows, Fig 4A and 4D), with some exceptions for NeuN-negative and GFAP-positive astrocytes (arrowheads, Fig 4A and 4D). This finding clearly indicates the absence of astrocyte specificity for the 0.3-kb cjGFAP promoter in the mouse cerebral cortex. A plausible explanation for the absence of astrocyte specificity is that the 0.3-kb cjGFAP promoter is too short and lacks the region necessary for suppressing neuronal expression. Although it may be less likely, another possibility is a mismatch between the species of the promoter (marmoset) and the integrated cells (mouse). We tested the latter possibility by examining the astrocyte specificity of the homologous 0.3-kb mouse-derived mGFAP as well as human-derived 0.3-kb hGFAP promoters. Lentiviral vectors expressing GFP under the control of the 0.3-kb mGFAP or hGFAP promoter were injected into the mouse cerebral cortex. Immunohistochemistry of the cerebral slices made 1 week after viral injection showed GFP expression specifically in the GFAP-positive and NeuN-negative astrocytes (Fig 4B and 4C), when mGFAP promoter, but not hGFAP promoter, was used. A quantitative analysis showed that more than 80% of the GFP-positive cells were astrocytes in the mouse cerebrum expressing GFP under the control of the mouse-derived mGFAP promoter (328 cells examined from 3 mice). In contrast, the ratio of GFP-positive astrocytes was only approximately 20–30% of all GFP-positive cells in the mouse cerebrum expressing GFP under the control of the marmoset- or human-derived GFAP promoter (402 and 330 cells examined from 3 mice, respectively) (Fig 4E). There was a statistically significant difference in the astrocyte specificity between the mGFAP promoter and cjGFAP promoter or hGFAP promoter (***p<0.001), suggesting a possibility that the loss of astrocyte specificity for the 0.3-kb cjGFAP promoter in the mouse cerebral cortex was attributed to a difference between the species.

### Retention of astrocyte specificity for the 0.3-kb cjGFAP promoter in the marmoset brain

**Fig 5:**
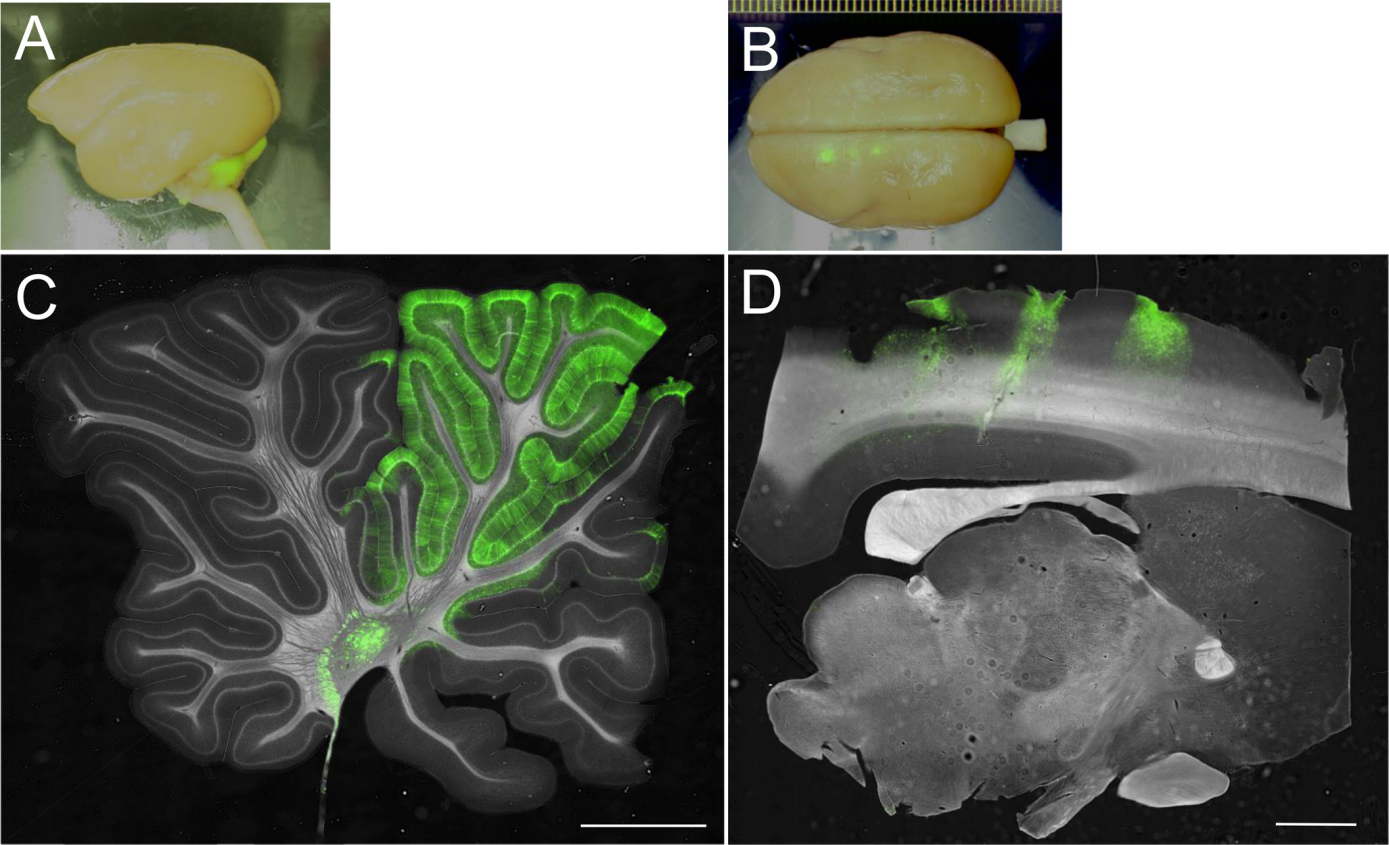
AAV9 vector-mediated GFP expression in the marmoset brain. AAV9 vectors expressing GFP under the control of the 0.3-kb cjGFAP promoter were injected into the cerebral and cerebellar cortices. (A and B) Bright field images of the marmoset whole brain overlaid with GFP fluorescence. (C and D) Bright field images of the sagittal sections of the cerebellar (C) and cerebral (D) hemispheres are presented with GFP fluorescence. Scale bars, 2 mm.

**Fig 6:**
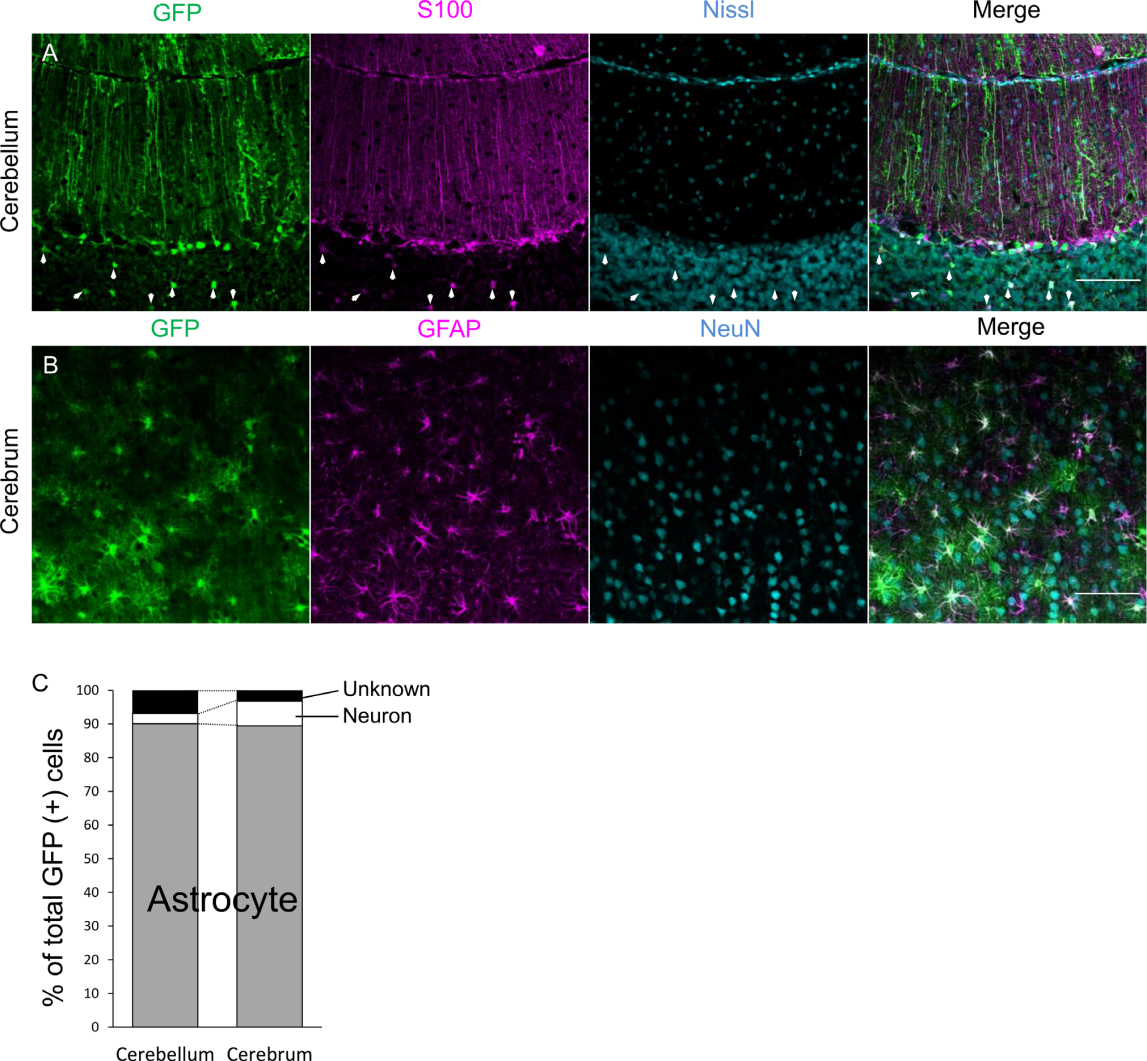
Astrocyte-specific GFP expression in the marmoset brain by AAV9 vectors carrying the marmoset-derived cjGFAP promoter. (A) Marmoset cerebellar slices virally expressing GFP under the control of the 0.3-kb cjGFAP promoter were triple-immunostained for GFP (green), S100 (magenta), and Nissl substance (cyan). (B) Marmoset cerebral slices virally expressing GFP under the control of the 0.3-kb cjGFAP promoter were double-immunostained for GFP (green), GFAP (magenta), and NeuN (cyan). (C) More than 150 GFP-positive cells in marmoset cerebellar and cerebral slices triple-immunolabeled for GFP, Nissl (cerebellum), or NeuN (cerebrum), and S100 (cerebellum) or GFAP (cerebrum) were randomly selected. The ratios of S100- or GFAP-labeled astrocytes and Nissl- or NeuN-labeled neurons to all GFP-positive cells were determined. GFP-positive cells without labeling for both S100 (or GFAP) and Nissl (or NeuN) were classified as ‘Unknown’. Note that approximately 90% of GFP-positive cells in both the cerebellar cortex and cerebral cortex were astrocytes. Scale bars (A, B), 100 μm.

To verify the astrocyte specificity of the 0.3-kb cjGFAP promoter in the marmoset brain, we used AAV serotype 9 (AAV9) vectors because AAV9 vectors have the potential to transduce significantly broader areas of the brain than lentiviral vectors [8] and, thus, are suitable for transduction in the larger marmoset brain. AAV9 vectors expressing GFP under the control of the 0.3-kb cjGFAP promoter were injected into both the cerebellar cortex and cerebral cortex of 1.8- and 2.4-year-old marmosets. The GFP expression profiles of the marmoset brains were examined 2–4 weeks after the viral vector injections were performed. Bright GFP fluorescence was observed in the cerebellar and cerebral hemispheres in regions surrounding the injection sites (Fig 5A and 5B). Sagittal sections of the brains confirmed robust and efficient GFP expression in the cerebellar and cerebral cortices (Fig 5C and 5D). To examine the cell types expressing GFP, the sections were triple-immunostained for GFP, Nissl substance and S100 (cerebellum) or GFAP (cerebrum). In the cerebellum, GFP was almost exclusively expressed in Bergmann glia (Fig 6A) and S100-positive astrocytes in the granule cell layer (arrows, Fig 6A). Similar to the cerebellar cortex, GFP was detected in GFAP-positive astrocytes in the cerebral cortex (Fig 6B). A quantitative analysis of more than 150 cells from both the cerebellar and cerebral cortices of 2 marmosets showed that approximately 90% of GFP-positive cells were astrocytes, whereas <10% were neurons (Fig 6C). These results show that, when introduced with viral vectors, the 0.3-kb cjGFAP promoter serves as an astrocyte-specific promoter in the marmoset cerebellar and cerebral cortices.

## Discussion

A series of previous transgenic studies examining the human GFAP promoter revealed critical roles for the B region and contiguous C1 segment in regulating the strength of GFAP promoter activity and astrocyte specificity [15–21]. The 5’ deletion of the original 2.2-kb GFAP promoter to 1.7 kb did not substantially compromise the promoter strength in U251 cells, a human glioma cell line that strongly expresses GFAP, whereas further deletion to approximately 1.5 kb, which removed the B and C1 regions, resulted in a drastic reduction in the promoter strength to 10% of the original 2.2-kb promoter [16]. Consistent with this finding, in our study, 5’ deletion of the cjGFAP promoter from 2.0 kb to 1.6 kb, which still carried the B and C1 regions, had little influence on promoter activity. However, deletion to 1.4 kb, thus removing the B and C1 regions, resulted in a drastic reduction in the promoter strength to <20% of that of the 2.0-kb cjGFAP promoter. Interestingly, the astrocyte specificity of the cjGFAP promoter was preserved for fragments of 0.3 kb or more in the mouse cerebellar cortex. Moreover, the 0.3-kb promoter showed significantly higher promoter strength than the 0.6-kb and 1.4-kb promoters, indicating that the 0.3-kb promoter was superior to the 0.6-kb and 1.4-kb promoters in terms of promoter strength and accommodation capacity for a transgene.

In the cerebral cortex, however, the 0.3-kb cjGFAP promoter also drove transgene expression in pyramidal neurons. There are at least 2 possibilities that could account for the loss of astrocyte specificity for the 0.3-kb cjGFAP promoter in the mouse cerebral cortex. The first possibility is that the deleted region upstream of -0.3 kb may be crucial for suppressing non-astrocyte expression. The other possibility is related to the use of the marmoset-derived promoter in the mouse cerebrum. Specifically, the species mismatch may explain the loss of astrocyte specificity for the 0.3-kb cjGFAP promoter. Initially, we supposed that the first possibility was more likely than the second. Thus, to exclude the second possibility, we cloned the homologous 0.3-kb GFAP promoter from the mouse genome and examined the promoter’s characteristics in terms of its astrocyte specificity in the mouse cerebrum. Unexpectedly, the 0.3-kb GFAP promoter of mouse origin specifically transduced astrocytes, thus suggesting the second possibility was correct. This result motivated us to test the astrocyte specificity of the 0.3-kb cjGFAP promoter in the marmoset brain. We then injected AAV9 vectors expressing GFP under the control of the 0.3-kb cjGFAP promoter into the marmoset cerebral cortex and subsequently observed astrocyte-specific expression of GFP in the cerebral cortex, as well as in the cerebellar cortex, which was similar to the expression pattern for the mouse GFAP promoter in the mouse brain. Therefore, the 0.3-kb cjGFAP and mouse GFAP promoters are thought to contain sequences that are critical for suppressing promoter activity in cells other than astrocytes. Moreover, the putative sequences are likely species-specific, at least in the cerebral cortex, but the sequences may be shared by mouse and marmoset cerebellar cortices, since the astrocyte specificity of marmoset-derived 0.3-kb cjGFAP promoter was preserved in the mouse cerebellar cortex. Further study is needed to clarify the mechanism that suppresses non-astrocyte expression of the GFAP promoter in different brain regions.

The packaging capacity for recombinant AAV vectors is approximately 4.7 kb, including the 2 inverted terminal repeats (ITRs). Because the total length of the 2 ITRs of AAV9 vectors is 372 bp, there is a capacity of approximately 4.3 kb for foreign DNA that can be accommodated between the 2 ITRs. Moreover, the woodchuck hepatitis post-transcriptional regulatory element (WPRE) [22, 23]–polyadenylation signal element is approximately 0.7 kb long. If a reporter gene such as GFP were to be co-expressed, it would require another 0.7 kb. Thus, the maximal allowable cDNA length is approximately 2.9 kb, including a promoter sequence. Thus, an efficient short promoter is valuable for the AAV vector-mediated expression of a large cDNA. The 0.3-kb GFAP promoter that we identified in this study allows us to express a transgene of up to 2.6 kb of cDNA together with GFP specifically in astrocytes. Our results identified a very short astrocyte-specific promoter region that extended the usability of AAV vectors for astrocyte-specific transgene expression in the marmoset brain.
